# Ontario Sets a Bad Example

**Published:** 1897-12

**Authors:** 


### ONTARIO SETS A BAD EXAMPLE.

In a recent report of a meeting of a veterinary society in New England, the Secretary wrote at the end of his minutes: "Thank Heaven, the everlasting subject of tuberculosis did not come up!" And it is to be feared that many veterinarians look upon discussions and articles on this subject in the same light. We should remember, however, that the matter of tuberculosis is one of the most urgent that is now confronting the live-stock industry and the veterinary profession, and is far from settled. The measures to be adopted in suppressing this widespread, destructive, and dangerous disease are not decided upon. Eminent authorities disagree as to the best methods to be employed, and the regulations of the different States and countries are varying and conflicting. This is a subject in which there is more interest among the laity and among the members of the medical profession than any other with which veterinarians deal. The difficulties to be met in planning and enforcing a campaign against tuberculosis of cattle are numerous and of the most varied character. *All of them call for the application of a sound knowledge of pathology and comparative medicine in general, and of this disease in particular.* The occasion for such knowledge will always be great, but in this formative period of the campaign it is more urgent than it will be at a later time, when plans have been tested and there is a general consensus of opinion as to those that are most practical, efficient, and best. The relations of this disease to the future of the live-stock industry and to the public health are the factors that should predominate in these considerations, and these factors are best understood by men trained in comparative pathology and in veterinary science. To delegate either the origination or execution of measures directed against the spread of tuberculosis to men not so trained is not only an injustice to the taxpayers and to the animal industry, but it is a crime against the consumers of cattle-products.

These remarks are incited by a perusal of a special bulletin on tuberculosis in cattle that has recently been issued by the Ontario Department of Agriculture. The authorship of the bulletin is not indicated; but this is unimportant, because it is almost entirely a compilation from the writings of well-known authorities on this subject, and a statement as to the provision made by the Ontario

Department of Agriculture for the testing of herds with tuberculin. It is to be noted that the Department of Agriculture has employed an agent to give "demonstrated instructions concerning the detection and treatment of tuberculous animals to those who require his services. He will attend meetings called by farmers' institutes and deliver addresses on this subject, and give full instructions concerning the use of the instruments, fluids, etc., that are used. He will at all times be prepared to properly test animals."

It is a grave question as to whether it is fair to the live-stock industry to give popular instruction concerning the use of the tuberculin-test. The general consensus of opinion among veterinarians and those who understand this subject best is against the giving of such popular instruction. *It should be distinctly understood that it is not a narrow or selfish view that leads to this opinion.* Do surgeons give popular instruction on hip-joint amputations and laparotomy to the laity? Do physicians inform their patients how to make Widal's test for typhoid fever, and how to produce diphtheria antitoxin? And does the shipmaster instruct his passengers how to pilot a vessel into New York harbor? It is not a narrow or selfish view that prevents the giving of such instruction. The average man is incapable of appreciating it, and if he were taught to believe that he possessed full information in reference to these subjects his knowledge would do himself and others far more harm than good. The Ontario Government is the first to attempt risky instruction of this character. There can be no doubt that farmers should be informed as to the nature of tuberculosis and the general means which they can employ to prevent it. But they should not be encouraged to apply the tuberculin-test themselves, for blunders will be the inevitable result. Many mistakes will occur. After a cow is once tested she cannot be tested reliably a second time until a long period has elapsed. Under such conditions the tuberculin-test is bound to fall into disrepute, and the benefits to be derived from an agent that furnishes the most promising means of eradicating tuberculosis will be lost to many communities.

It is amazing and alarming to note that the instruction in reference to tuberculosis and the use of tuberculin is not given by a veterinarian familiar with the intricate points of animal pathology and the many essential technical questions involved, but by a certain lieutenant-colonel, who has presumably made a more or less careful study of this single disease, but cannot possess the full knowledge of other diseases and collateral subjects that are absolutely indispensable to a proper conception of this question in all its important bearings.

Ontario has a high reputation as a breeding-district. The live-stock show held annually in Toronto is the best on the Continent, and cattle from Ontario are sent out to improve herds in all parts of Canada and the United States. Do those responsible for the protection of the live-stock industry in Ontario appreciate the gravity of the action they have taken in reference to tuberculosis of cattle? Do they not know that an intelligent interest in this disease is growing rapidly everywhere, and that purchasers are becoming more strict in their requirements from day to day; and do they not know that such teaching from such a source will lower confidence in them and will react to the injury of their industry? If the farmers of Ontario are taught and encouraged to make an indiscriminate use of the tuberculin-test, how can purchasers of cattle there know that their animals are free from tuberculosis, even if subsequently tested by experts?

The tuberculin-test should be under some competent, reliable supervision, and should be reported to some central authority. This is the opinion of the highest veterinary body on the Continent, as the following resolution adopted by the 1897 meeting of the United States Veterinary Medical Association is proof:

*Whereas*, the tuberculin-test has been proven to be the only reliable ante-mortem means yet discovered of determining the existence of tuberculosis in its obscure forms; and,

*Whereas*, the repetition of this test on the same subject tends to lessen the characteristic reaction, thus producing a non-responsive condition of the animal, which may be mistaken for soundness; and,

*Whereas*, these facts may be, and are, taken advantage of by the irresponsible and unscrupulous to aid in the disposition of diseased animals; be it

*Resolved*, that the private employment of this test, except as supervised by competent and responsible persons, is fraught with danger to the public.

Another phase of this subject should be of interest to the Ontario authorities. Ontario has always been looked to as a veterinary centre, and numerous students have been drawn to her veterinary college. It is a sorry reflection on this college to place a layman in charge of such an important branch of veterinary work in the province in which it is situated, and will be apt to cause a false impresssion regarding it.

---

ON another page of the JOURNAL will be found a fitting tribute to the memory of our esteemed fellow-worker, Dr. W. H. Harbaugh, written by one who knew him intimately and well. It is a just encomium to a rare and tireless worker in our profession, and we join most heartily in the wish of the writer, that Dr. Harbaugh's example to his fellow-men may be a rich influence in moulding the career and efforts of others to serve their chosen profession as well.

---

Among our reports of cases in this issue will be found one of peculiar and important surgical interest. Only those who are fully conversant with the difficulties of control and asepsis in the lower animals, the equine family especially, can fully appreciate this triumph in abdominal surgery. In our October number were recorded several interesting and successful surgical operations by the same writer, and they reflect great credit upon our esteemed colleague, Dr. E. Mayhew Michener, and place him in the front rank of surgeons of comparative science in abdominal surgery. These successful issues in major surgery open up a field of great value in equine, bovine, canine, and porcine surgery which must add greatly to the successful preservation of many like cases in valuable breeding-stock which in the past have usually died or were rendered comparatively worthless for indefinite periods.

---

Rochester, N. Y., opens a new field of association work, and we are very sure it will be felt as a potent influence for good in the Empire State. The society will include the members of the profession from several adjacent counties, and the very representative names of the profession in Rochester who are directing this movement give assurance of its success. We welcome this organization to the field in New York State because our profession needs better legislation in some directions, and some that has already been gained needs modifying, while other lines of work out of the province of the veterinarian will never be successful until they are directed and performed by veterinarians, of whom New York State has many able representatives.

